# Editorial for the Special Issue on Self-Assembly of Polymers

**DOI:** 10.3390/mi10080519

**Published:** 2019-08-05

**Authors:** Anna S. Vikulina, Dmitry Volodkin

**Affiliations:** 1 Fraunhofer Institute for Cell Therapy and Immunology, Branch Bioanalytics and Bioprocesses, Am Mühlenberg 13, 14476 Potsdam-Golm, Germany; 2 School of Science and Technology, Nottingham Trent University, Clifton Lane, Nottingham NG11 8NS, UK

The self-assembly of polymers is a powerful tool for producing various functional materials with a high precision from nano- to macroscale. Nowadays, the polymer self-assembly has become extremely attractive for both biological (drug delivery, tissue engineering, advanced cell culture) and non-biological (packaging, semiconductors) applications. Besides this, a number of key biological processes in nature are driven by self-assembly of macromolecules.

This special issue gives insights into diverse spectrum of peculiar tailor-made self-assembled polymer structures and hybrid materials that have been designed through modern powerful techniques and approaches such as the micellization [1], hydrogel formation [2,3], self-assembly at the air-liquid interface [4], the layer-by-layer deposition and the hard templating [5,6], solvent evaporation [7,8], photo-induced [9] and enzyme-mediated cross-linking [10].

In particular, significant attention has been attracted to the layer-by-layer assembly of biopolymers. Thus, Balabushevich et al. have presented the study of mucin-based layer-by-layer assemblies that are mainly composed from the natural component of mammalian mucus and therefore show high potential for mucosal drug delivery [6]. In the recent years, collagen is gaining interest as a component of modern biomaterials [2]; some novel sources of the biopolymers to be used in the self-assemblies, e.g. biopolymers of marine origin, have also been described [2]. Yang et al. reviewed the current state of biocompatible and biodegradable polypeptide-based nanomaterials [9].

Another issue in the field of polymer self-assembled structures that has been reflected in this special issue is the design of stimuli-responsive materials. This includes both, chemical stimuli such as pH and physical stimuli including light. In this special issue, Xu et al. presented an elegant way for fabrication of pH-responsive amphiphilic block copolymer-based assembles [1]; Yang et al. paid special attention on photo-triggered assembly of polymer-based nanostructures [9].

Bridging the gap between the science of the polymer self-assembly and the industrial application of this technology, Nelson et al. have designed and constructed a new solvent vapour annealing chamber that allowed the potential scaling-up of the production of block copolymer thin films [7].

The next equally interesting aspect that has been deciphered in this issue is organisation of self-assembled polymer-based and hybrid structures in 3D. Herein, microfluidics-assisted assembly of porous calcium carbonate templates and their covering with polymer coatings opened new avenues for the fabrication of tailor-made biocompatible scaffolds for advanced tissue engineering and 3D cell culture as reviewed in [3]. Surface patterning with the polymers [8] and crystalline colloidal monolayers [4] also have been demonstrated.

That is rather interesting to note that while the papers listed above mostly aimed at the invention and investigation of the new types of materials, the paper presented by Jeannot et al. took under the consideration a well-known poly(styrene-sulfonate)/poly(diallyldimethylammonium chloride) polyelectrolyte pair and the authors have shown principally new insights into the classical understanding of the internal structure of multilayer capsules [5]. Their findings potentially have high importance for the field of controlled drug release.

Finally, it is worth to emphasize the review of Savoca et al. that summarises the up to date knowledge on the biocatalysis by transglutaminases, enzymes that crosslink polymer-bound glutamine and lysine residues and plays a special role in the formation of extracellular polymer matrix [10]. The review highlights how bioinspired polymer cross-linking with transglutaminase allows fabrication of biocompatible scaffolds and hydrogels for biotechnological and bioengineering applications.

We would like to take the opportunity to thank all the authors for submitting their papers to this special issue. We also want to thank all the reviewers for dedicating their time and helping to improve the quality of the submitted papers.
